# Cognition of agriculture waste and payments for a circular agriculture model in Central China

**DOI:** 10.1038/s41598-020-67358-y

**Published:** 2020-07-02

**Authors:** Haimanot B. Atinkut, Tingwu Yan, Fengyi Zhang, Shengze Qin, Hao Gai, Qiqi Liu

**Affiliations:** 1 College of Economics and Management, Huazhong Agricultural University, Wuhan, 430070 China; 2 Hubei Rural Development Research Center, Wuhan, 430070 China; 3 College of Agricultural and Environmental Science, University of Gondar, 196, Gondar, Ethiopia

**Keywords:** Psychology, Environmental social sciences

## Abstract

An integrated model combining multi-layer cradle to cradle approach: cost effective, technically sound, and bioenvironmental cutting-edge agricultural waste assessment technologies are lacking; to address this gap, the study proposes circular agriculture model (CAM) to support an integrated, bio-based, sustainable and broadly applicable rural society. CAM is an innovative, quasi-public product, bio-commodity, and concept. This study uses survey data on the Chinese province of Hubei to consider the returning of crop residues to the soil and manure for compost or biogas production (first-generation biorefinery). It explores farmers' environmental understanding and their willingness to pay (WTP) under a “polluter pays” principle. Factors, including education, infrastructure, trust in family-neighbors, and environmental attitudes, have a significant effect on WTP. Moreover, income, sustainability-recycling behavior, environmental perception, perceived usefulness-easiness, and trust-in-government positively affect farmers’ WTP, whereas environmental attitude, intention, and selfishness have a negative effect. It, therefore, calls for the integration and cooperation of private, government, business, R&D and public welfare to value the combined rural traditions, religion, philosophy and belief, socio-psychological and altruistic values of local communities, which are essential for building trust and providing ecological security, tech spill-over, thereby indirectly helping farmers to restore their livelihoods.

## Introduction

Unprecedented challenges of today’s society include distribution of inclusive food system, water—energy security, resource depletion, climate change adaptation and mitigation cost, gender equality, economic crisis, waste management, over-reliance on non-renewable-energy sources—fuel and gradual transition from coal to-bio-based renewable energy sources such as bio-refinery from biomass. In this context, we need to rethink the future sustainable development, in particular agricultural development, to address resource constraints, sustainable food production and consumption, environmental concern, inefficiency, and sustainability. Agriculture is a chief source of food and waste, the production of greenhouse gases, and the energy supply of biomass.

In China, agricultural development has led to the country’s substantial growth over the past three decades^1^, responsible for around 11% of national greenhouse gas (GHG) emissions, with cereal production accounting for a large proportion^2^ (about 32%) and crop residues harbor the promise of meeting the future Chinese renewable energy requirements^,^^3^. Recently, agricultural waste such as crop residues and straw, bran, husks, manure, and so on increasingly used for biogas—biofuels production^4^. Rice is a staple crop in many Asian, and the world’s third-largest grain crop next to wheat and corn^5^. Globally, some 973.9 Mt of cereal crop straw and residues rice straw and husk are produced per year^6^; China is an agriculture-based country rich in crop biomass and the largest producer of rice straw accounting 180–270 tons per year^7^. A large proportion of rice straw burns on the field, for example, in India, produces about 25% surplus of rice straw burns on an open field and accounts 0.05% greenhouse gas emissions^8^. Bioeconomy is, therefore, a solution.

Bioeconomy has become a significant policy priority agenda^9^. Early-circular bioeconomy countries include the United States, Finland, Germany, the Netherlands, China, Korea, Japan, and South Africa. Biorefineries have the ability to minimize the dependence on crude oil by using agricultural waste sources such as straw, husks, and stovers from corn and rice residues^10^. Bio-based biomass energy meets the growing demand for renewable energy^11^. To meet the demand of bio-based system engineering experts in the field of circular bioeconomy, bio-refinery, and bio-based process bio-based bio-fuels and bio-commodities, six higher educational institutions among China and Europe signed an agreement to work together by 2016^12^.

Recently, biofuels and biochemicals production has shifted from food and non-food^13^ to the use of first-generation biomass such as corn, sorghum and sugarcane^14^, Lignocellulosics (second-generation biomass) and seaweeds (microalgae, third-generation biomass)^15^. However, the second and third-generation of biorefinery platforms have a complex structure that is economically unworkable, technical unsuitable, and less generally applicable at the farm level. Circular agriculture is therefore strongly recommended and fills the gaps of biorefinery and other circular bioeconomy innovations and activities in a wider agricultural society that is a cradle to cradle approach.

China has been successful in solving many of the challenges in agricultural production; however, there remain enormous hurdles, such as sustainable food—energy security, and sustainably becoming a low carbon economy. Following the socio-economic transition in China that led to the expansion of urbanization and industrialization, thereby resulting in environmental pollution^16^, rural areas face multiple ecological problems^17^. As highlighted in recent studies^18^, in the fall of 2013, President Xi Jinping proposed the One Belt and One Road (OBOR) initiative to stimulate new environmental risks awareness across the entire Eurasian continent, especially in countries with poor ecological governance records such as former Soviet republics and Russia^19^. In response to environmental problems: since 2002 the Chinese government has promoted a sustainable development model, the “circular economy”, “ecological restoration” and “ecological civilization”, “Green GDP”^20^. The Ministry of Ecology and Environment (MEE) was formed in 2018 to develop low carbon development activities in line with circular agriculture (CrA). Most recently, China has emphasized on biological and sustainable waste management methods, for example, cockroach farming^21^ and AI-trash deign to stop food waste^22^. Thus, CrA works in a closed loop of sustainable agriculture production. CrA (healthy-agroecology) promotes local food systems and sustainable livelihoods in Africa and helps to meet the target of SDGs targets^23^.

This study proposes a circular agriculture model (CAM)—Circular agriculture (CrA) is the facets of circular bioeconomy, which aims to solve the challenges of the farm-based rural economy and environmental problems. CAM is an eco-agricultural innovation promoting a multi-grade use of biocommodities and byproducts. Thus, multilevel-recycling and reusing agriculture waste means turning all waste into an energy resource and production. Hubei was a pilot promotion province for circular agriculture development (CAD)—located at the heart of China, has huge agricultural potential as the hub of technology and science. Hubei has a large volume of agricultural waste generation. Hence, introducing sustainable agricultural technology and practices is highly demanded.

Evidently, no study has examined agriculture waste recycling in light of the behavioral and economic theories in CrA development and assessed farmers' willingness to pay (WTP). This study bridges this gap. The demand for CrA is expected to grow, and very little is known about farmers’ attitude and intention towards recycling and free-riding of common products, such as CrA. Thus, this study examines farmers' knowledge of the environmental impact of agricultural waste and, where a hypothetical market valuation is necessary, their WTP for recycling. CrA is a quasi-public good. For an assessment of environmental public good, the choice is either choice experiment or contingent valuation. The contingent valuation method (CVM) is a widely used technique for hypothetical market scenarios^24^. Contingent valuation (CV) surveys are employed to elicit bid preferences for CrA technology (i.e., manure for biogas and returning crop residues to soil).

We contribute to the literature by introducing particular agriculture concept designs that consider local agro-climatic, cradle to cradle approach, and traditional values during CrA policy drafting, technology design and promotion; link agriculture, industry, and people together; understand traditional foundation of rural people; and integrate different theories, such as planned behavior, technology acceptance, innovation diffusion, and environmental psychology in promoting CAM.

The objectives of this article include recognizing farmers' knowledge and disposal culture of agriculture waste, examining factors affecting farmers’ WTP for CrA system, and calculating farmers’ WTP amount regarding agriculture waste recycling. This article is organized as follows. The next section presents results. The third section illustrates the discussion, and the fourth section presents the methodology, followed by concluding remark. Acronyms and /or abbreviations such as CAD, CAM, CE CrA, CV, CVM, WTP and WUR shall be used in this article.

## Results

### Households’ characteristics and infrastructure

**Figure 1:**
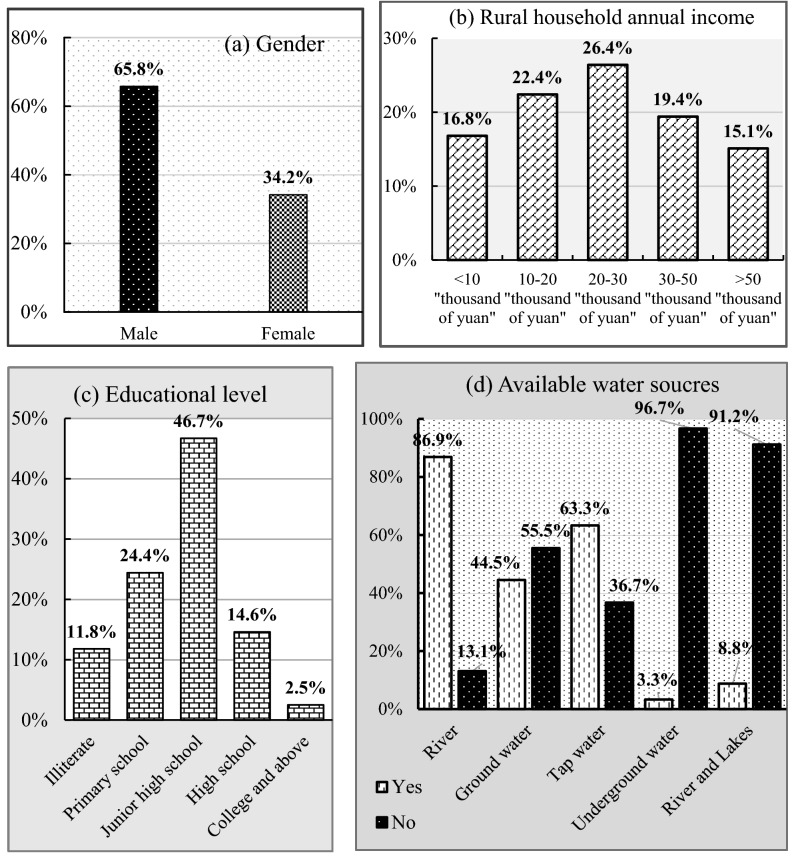
Basic characteristics and infrastructure.

Figure 1 illustrates some characteristics of households and infrastructure facilities. Householders who are working outside the home are considered males. Surveyed householders reported that 34.2% are females (Fig. 1a). Women are close to the environment, and men are near to information and play a decision-making role in environmental management. The average age of householders is about 52 years old, with a minimum of 23 and a maximum of 91 years old. As farmers' age increases, they expected to have adequate experience in environmental management. The average farming experience of respondents is approximately 32 years with a minimum of 0 and 70 years of farming. Concerning farmers' educational level, 11.8% are illiterate, 24.4% attended primary school, 61.3%, junior high school, technical, and vocational, and 2.5% reached college or university (Fig. 1c). In terms of farmers' annual income, the average yearly income is 31.05 thousand yuan (total households’ annual income = 31.05 × 398 = 123.579 million). Farmers’ with annual income equaling ten thousand were less than 16.8%, those their annual income between the range 10–20, 20–30, 30–50, and above 50 thousand yuan were 22.4%, 26.4%, 19.4%, and 15.1%, respectively (Fig. 1b). Householder annual expenditure was 29 thousand yuan; of this, 20% were agricultural expenditure. During the survey period, majority of householders replied that they could access water supply. Locally available water sources and respondent’s responses are rivers (86.9%), groundwater (44.5%), tap water (63.3%), underground water (3.3%), river (8.8%), and lakes (see Fig. 1d). In 2012, rural male residents of Hubei province accounted^25^ for 51.30%. Thus, the study samples are representative of the Hubei province.

### Waste disposal experience

The result indicates sound rural agricultural waste handling, and environmental protection has been carried out in Hubei province. This situation indicates that farmers have better cognition of agricultural waste impacts on the environment (see Table 1).

**Table 1:** Waste disposal experience.

Attributes	#	%
**Central treatment**		
Yes	165	41.5
No	233	58.5
**Domestic garbage**		
Free throw	139	34.9
Put in an exclusive garbage collection	158	39.7
Landfill	100	25.1
Pay for a recycling bin	1	0.3
**Domestic wastewater treatment**		
Through a special sewer discharge	66	16.6
Directly discharge into the surrounding environ	308	77.4
Other	24	6.0
**Toilet manure**		
Rivers and lakes	8	2.0
Farmland	263	66.1
Biogas pool	103	25.9
Other	24	6.0

Farmers were asked of inputs and chemical use trends. Of the surveyed farmers, 20.6% use fertilizer, and 12.1% use pesticides to the recommended standard shown in Table 2. It implies that farmers in the study area over- or under-apply the recommended dose of farm inputs and chemicals. Thus, there is scope for formula fertilizer, green fertilizer, compost, and residue return to soil regarding future sustainable agricultural technology, science, and development. Surveyed farmers' fertilizer and pesticide usage are in line with CrA principles. Thus, there is a good practice of modern agriculture in Hubei.

**Table 2:** Farm chemical use.

Variables	#	%
**Fertilizer application to the standard**		
Yes	82	20.6
No	316	79.4
**Pesticide application to the standard**		
Yes	48	12.1
No	350	87.9

**Figure 2:**
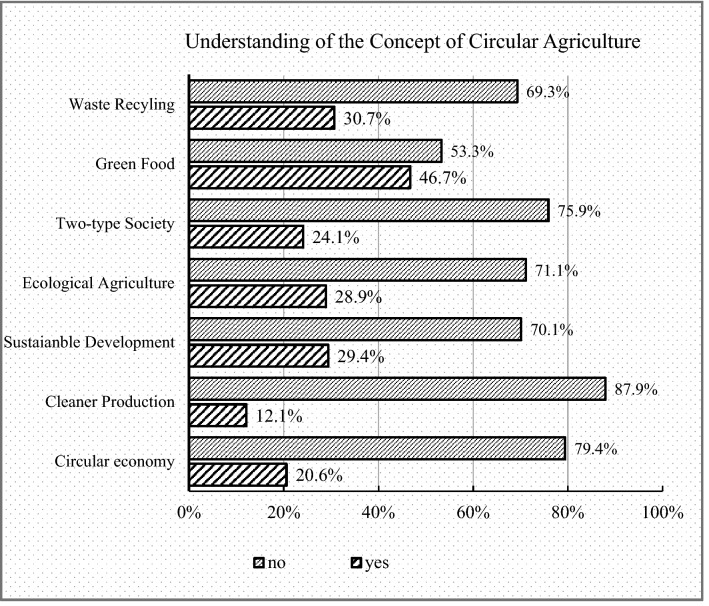
Understanding of the concept of CrA.

According to Ellen MacArthur Foundation^26^ describes CrA as a segment of circular economy (CE), where sustainable development aids meet sustainable development goals. Surveyed householders posed questions to measure their level of understanding of CrA and sustainability. Figure 2 illustrates farmers' response to their knowledge of the typology of CAM and innovation recycling practices.

### Environmental concern

**Figure 3:**
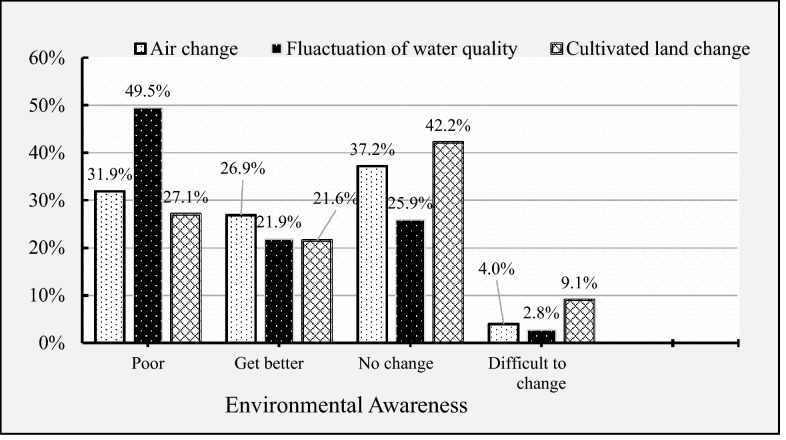
Environmental awareness.

Figure 3 illustrates the farmers' level of environmental concern. Environmental cognition leads to the development of environmental attitude and behavior. The environmental perspective is a step by step process from awareness, perception, and building attitude. This implies that farmers have a better understanding of local environmental dynamics in connection to air, water, and land.

**Figure 4:**
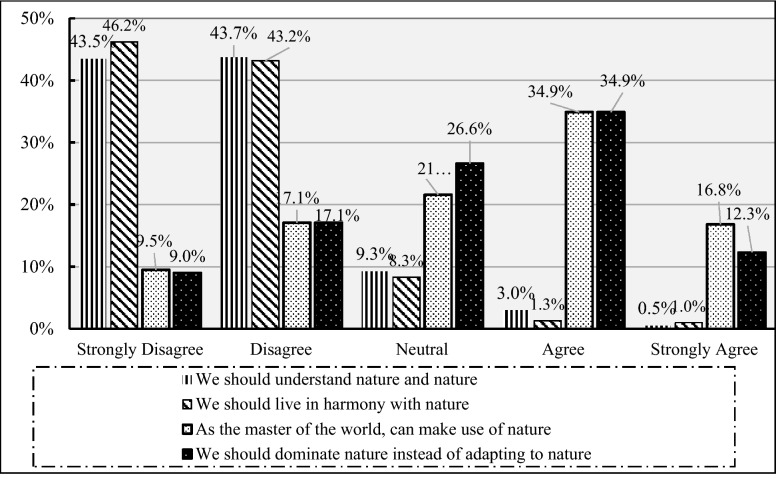
Altruism and environment.

Taoism and Confucius's teachings accord with living in harmony with nature. When anthropogenic interaction with the environment is not a win–win, it harms the environment. Batson and Powell’s social exchange theory^27^ against the egoism of self-benefiting results in continuous improvement in mutual benefits, empathy, and prosocial behavior, such as helping comforting, sharing, and cooperation^5^. These traits motivate the increase of one's welfare against egoism. Thus, Comate’s goal is ant-egoism. Figure 4 illustrates the degree of farmers' unselfishness to environmental conservation and protection and their appreciation of the well-being of future generations. It implies that circular agricultural science and technology have not had an immediate economic benefit; thus, altruism values may be lower, and higher-income farmers may be more altruistic.

### Analysis of willingness to pay responses

In standard contingent valuation, survey results can be used to determine whether farmers are willing to pay for waste recycling technologies. The coefficients estimated to obtain mean WTP values are shown in Table 3. Our analysis suggests that protest zeros should remain in the sample and treated as true zeros, because those individuals who are willing to pay also hold significant protest beliefs that influence their decisions. The mean WTP is 11.25 yuan per month, with a minimum zero WTP above 90 yuan. The standard expected to pay can be computed as:

$$\begin{aligned} & 11.25\, \times \,12\,{\textit{months}} = 138\,yuan\,per\,household\,per\,year \\ & {\text{The}}\,{\text{minimum}}\,{\text{WTP}}\, = \,138\, \times \,75.38\% \, = \,104.02\,yuan\,per\,year \\ & {\text{The}}\,{\text{five-year}}\,{\text{maximum}}\,{\text{expected}}\,{\text{WTP}}\, = \,138\, \times \,4\, = \,552\,yuan\,per\,household. \\ \end{aligned}$$

**Table 3:** WTP bid values.

Bid (WTP value/yuan)	# (person)	%	Cumulative (%)
0	148	37.2	37.2
10	174	43.7	80.9
20	46	11.6	92.5
30	19	4.8	97.2
40	5	1.3	98.5
50	6	2.0	99.8

### Factors affecting willingness to pay responses

As discussed, variables including gender, age, education, infrastructure, knowledge of CrA, infrastructure, trust-in-family, interpersonal trust, perceived control to environment, environmental attitude towards the labor market, attitude towards health impacts, and environmental pollution are assumed to affect farmers WTP for CrA (see Table 4). The coefficient of respondents’ gender is positive and statistically significant at the 1% level. More specifically, male householders have a higher WTP for CrA, which may be due to male householders having better access to information on the environmental. However, as Elliott^28^ notes, women have a positive impact on green consumption, which contradicts our result. The age of farmers is statistically significant at the 5% probability level and negatively affects farmers' WTP for CrA. For every unit increase in age, the householder’s WTP tends to decrease. Aging farmers are expected to have a better experience than young farmers but are conservative toward the adoption of new technology and have less WTP; our results agree with Morris and Venkatesh^29^. The educational level is statistically significant at the 1% level and positively affected farmers' WTP for CrA. The higher the education level of a householder, the higher the WTP for CrA, all other factors being equal. Zhang and Paudel confirmed that education activities had significant positive effects on the behavior regarding resource conservation^30^, pesticide use in Punjab^31^, and green consumption^28^, which is also consistent with our results.

**Table 4:** Determinants of WTP.

Variables	Coefficient	SE	*t* statistics	Marginal effect
Gender	0.91187	0.28595	3.19***	0.6583
Age	− 0.02842	0.01444	− 1.97**	51.9849
Education	0.56620	0.17692	3.20***	2.7161
Infrastructure	0.77034	0.30073	2.56**	0.7161
Knowledge of CrA	1.04147	0.31177	3.34***	0.4372
Trust in neighbors	1.31624	0.13472	2.35**	2.8116
Trust in family	− 0.34668	0.12678	− 2.81***	2.9824
Perceived control	− 0.27091	0.12335	− 2.20**	2.8719
Perceived likely	0.44282	0.12288	3.60***	3.1985
labor attitude	0.32811	0.12396	2.65***	2.8895
Health attitude	− 0.48780	0.18421	− 2.65***	1.7337
_Cons	− 0.70050	1.29245	− 0.54	
**Model goodness of fit**				
Obs			398	
LR chiz(11)			114.41	
R2			0.2575	

*, **, and *** indicate significance levels.

As expected, infrastructure is statistically significant at the 5% level and positively affects farmers’ WTP for CAD, implying that farmers having adequate infrastructural facilities since their WTP for CrA tend to be higher. The result on the Knowledge of CrA is significant at the 1% statistical level and positively affects farmers' WTP for CAM as expected. It means that, as farmers better understanding of CrA, their WTP for CAM is high. Farmers had high WTP for irrigated water regarding their knowledge of water services^32^, which is also consistent with Fryxell and Lo^33^. Trust in neighbors is statistically significant at the 1% level and positively affects farmers' WTP decisions on agricultural recycling technology. A unit increase in farmers' interpersonal trust, increases farmers’ WTP. It means that norm reciprocity is correlated with farmers' WTP. Farmers trust-in- family is statistically significant at 1% level and positively affects farmers willing to pay as expected. This means that as farmers believe in a family decision, their WTP decision to CA increases. Our results are in line with Dessart^34^.

Perceived control is statistically significant at the 5% level and negatively affects farmers' WTP for CrA. It implies that as farmers perceive low behavioral control, WTP decisions tend to high. Perceived likelihood of performance is statistically significant at the 1% level and positively affects farmers' WTP decision for CrA. It means that as farmers are more likely to use the recycling technology, their WTP for CrA is higher and vice-versa.

Attitude to labor supply is statistically significant at the 1% level and positively affects farmers' WTP for agricultural waste recycling technology. It means that as farmers have a positive attitude toward recycling technology labor consumption, their WTP is high. Farmers’ attitude towards recycling health benefits is statistically significant at the 1% statistical level and affects farmers' WTP for CrA. A unit increase in farmers’ recycling attitude to health benefits increases their WTP for CrA.

### Amount of WTP responses

Table 5 reveals signs, magnitudes, and the statistical significance of estimated parameters. Of 13 variables hypothesized to explain farmers’ WTP for CrA, 7 were found to be statistically significant at the 1% level. They participate in WTP bids, which is explained by a model efficiency of about 66%. The model includes environment attitude, environmental intention, sustainability behavior, recycling behavior, perceived easiness, and environmental selfishness.

**Table 5:** Outcomes of the Tobit regression.

Variables	Coefficient	SE	Z value	Marginal effect
Constant	0.04736	0.57055	0.08	
Annual income	0.07261	0.03481	2.09**	3.10512
Farm size	0.20759	0.10738	1.93*	1.44221
Recycling intention	− 0.48618	0.11348	− 4.28***	1.82915
Environmental intention	− 0.34214	0.11221	− 3.05***	1.88945
Sustainability behavior	0.53853	0.10087	5.34***	2.13065
Recycling behavior	0.26663	0.09120	2.92***	2.3191
Perceived usefulness	0.26725	0.08254	3.24***	2.83668
Perceived return	− 0.13909	0.09653	− 1.44	2.38693
Environmental perception	− 0.34190	0.11520	1.86 *	1.82663
Perceived easiness	0.16012	0.08605	3.82***	2.41457
Institutional trust	0.17233	0.07754	2.22**	3.08543
Trust in government	0.12550	0.06831	1.84**	2.59045
Environmental selfishness	− 0.36309	0.07399	− 4.91***	3.20854
**The goodness of fit measures**				
Lchi2(13)				94.23
Pseudo R2				0.0712

*, **, and *** indicate statistical significance at the 10%, 5%, and 1% levels.

Annual income is the net income of households in yuan per annum. Annual income is statistically significant at the 5% level and positively affects farmers' WTP for CrA. A unit increment in the annual income in ten thousand yuan increases farmers' likelihood to pay extra for CrA, all other factors being equal. It means that the coefficients of annual household income in a typical year are more likely to induce a household’s WTP. Our result is consistent with Khan and Damalas^31^. Farm size refers to the size of arable land in mu. Farm size is significant at the 10% statistical level and positively affects farmers’ WTP for CrA. The larger the farm size, the higher the WTP for CrA. This result confined Mu and associates^32^. Recycling intention is statistically significant at a 1% level and negatively affects farmers’ WTP. It means that if recycling technology design does not consider local climatic and agricultural conditions, farmers are less likely to pay. The environmental intention is significant at the 1% statistical level and negatively affects the WTP. It implies that if farmers believe agriculture waste recycling technology is a kind of environmental protection technology, they might pay less amount of money because environmental goods are less profitable in the short run.

Sustainability behavior is statistically significant at the 1% probability level and positively affects farmers' WTP. It means that as farmers possess sustainability behavior and perceive recycling technology in agricultural production and recycling agricultural waste as sustainable, they are more likely to pay the maximum bid value. Our result agrees with Wang and associates^35^. Recycling behavior is significant at the 1% statistical level and positively affects the WTP for CrA. This implies that, as farmers exhibit positive recycling behavior, they are more likely to pay for the provided bid amount.

Perceived usefulness is significant at the 1% statistical level and positively affects WTP. Famers recognize that agricultural waste recycling technology is easy to use through simple training; they are more likely to pay the maximum amount in ten thousand yuan. Economic benefits motivate recycling behavior^36^. Environmental perception is significant at the 10% statistical level and negatively affects farmers' WTP amount. If farmers have a lower recognition level of how recycling waste can cause less ecological pollution, they may be less likely to pay the maximum amount of yuan. Perceived easiness is significant at the 1% statistical level and positively affects farmers' WTP for CrA. It means that, as recycling technology is easy to learn and use, farmers may be more likely to pay for CrA.

Institutional trust is statistically significant at the 5% level and positively affects farmers’ WTP. If the government provides subsidies for agriculture waste recycling, farmers are willing to pay for CrA. This result is consistent with Putnam^37^.

Trust in governments is statistically significant at the 5% probability and positively affects farmers willing to pay as expected. As farmers trust in government subsidies and entities, they are most likely to pay for CrA. It means that if the village cadre tells them to recycle agricultural waste, they will agree to it.

Environmental selfishness is statistically significant at the 1% level and negatively affects farmers’ WTP. Farmers who have the wisdom to live harmoniously with nature are more likely to pay for CrA and vice-versa. It means that farmers who want to exploit nature instead of adapting to nature are negative and statistically significant and are less likely to pay for CrA.

The perceived economic returns are statistically insignificant and might not affect farmers' WTP, which was against our expectations. This result may be because recycling has no financial returns in the short run. Whereas farmers consider how recycling waste can quickly bring financial returns. The coefficients of monetary gains are not statistically significant. This may be because farmers adoption decision depends on short-term economic gains.

## Discussion

Circular agriculture (CrA) is an eco-environment and resource restoration concept and practice. CrA is a sustainable remedy for environmental problems emerging from agriculture. Though it is difficult to define CrA, it has binding principles that encompass climate-resilient, enhancing carbon sink and sequestration, improving soil health, improving crop-animal production, healthy nutrient cycling, and environmental protection^38^. The concept of CrA is a cradle to cradle^39^ thought and environmentally sound. CrA, as the key element of broader environmental management, would be much effective with volunteer programs and dedication individuals sacrificing for the future and the benefits of others are shown to be effective^27^. Moreover, CrA is in line with the 17 SDGs on environmental sustainability^40^, sustainable energy initiatives compatible with climate awareness^41^, and sustainable production^42^.

In China, antecedent 2007 CrA lagged in adoption and has since become part of the agricultural development policy. However, in 2013, in the 18th CPC meeting and 3rd agriculture census gets excel attention, president Xi Jinping stressed that “we must be the main provider of our food” by improving agricultural infrastructure and equipment and structural reform in agricultural side-supply, thereby optimizing agriculture products and increasing efficiency and competitiveness of the industry. Xi’s speech paid homage to sustainable modern agriculture production. Thus, study CrA is significant. CrA was soon promoted on pilot provinces, including Hubei. CrA is a novel approach to building a circular rural economy, while stimulating eco-efficiency and agricultural output growth^43^. A cropping pattern has both ecological and economic advantages^44^. For example, Yan-yan described CAM as an innovative sustainable agricultural development pattern^45^. Hua-jun in 2006 referred to CAD as the effectiveness of ecology, society, and economic development coupled with interaction, collaboration, and co-improvement at the level of targets, participant units, technology, and systems. Dai and Quanle confirmed that many elements play a role in the development of modern agriculture, such as market, industry, science, intensiveness, input service, and government^46^. However, such development is retrained by the lack of agricultural resources and agro-ecological environments to satisfy both local and international food markets^45^. A study on the Fujian province showed that the construction of ecological stewardship and rapid rural infrastructure development represent the foundation of CrA development^47^. CrA positively affects the ecology, especially in terms of rural energy, water, light, thermal energy, as well as biodiversity in agricultural biology^48^.

Recently, the Wageningen University & Research (WUR) organized a discussion series in collaboration with Brussels in late September 2018, “Mansholt lecture 2018”^49^. Generally, WUR connects CAM with farm animal and crop production, food system, soil health, climate change adaptation, and mitigation to organic farming^50^. Therefore, empirical shreds of evidence confirmed that CAM is alternative farming for low carbon economy and sustainability. Recent studies in china focused on CrA development and addressed global climate change^51^. CrA includes the cycling of material flow, a comprehensive endogenous and natural growth of efficient and orderly operation, continuity of the overall development, and promoting mutually beneficial co-ordination. Sustainable strategies for low carbon agriculture in a “*two-type society*”* is* an innovative product to build CrA for green agriculture development^52^. Studies show that the foundation of CrA—low carbon agriculture^53^—lessen global climate change and improve food security in South America^54^, reduces external input use in Macedonia^55^, and addresses rural climate change problems in China^56^. Moreover, deficient enforcement of relevant legislation and regulations and fundamental technologies for rural solid waste (RSW) treatment and disposal are serious problems remain^57^.

Recommending recycling by studying its adoption determinants is fundamental. However, several factors, such as essential household characteristics, interest, economic condition, cognition of local environment, perception, and attitude towards the environment, behavioral intention, and recycling behavior for sustainability challenges CrA promotion. Pro-environment protectionists suggested administrative measures to resolve pressing environmental problems, including “Carrot and Stick” or “reward and punishment”^58^. However, none of these waste management strategies function separately; instead, the Confucius thought of social harmony and cooperation is more effective^59^. More importantly, to encourage farmers to use sustainable CrA, innovative and modern agriculture technologies that reduces the application inputs (e.g. fertilizer and pesticide) in China, two theoretical suggestions were provided for the government; the first strategy was an economic incentive measure (green expenditure) and the second was a punitive measure, applying discouraging mechanisms, such as taxes and sewage charges^60^.

By contrast, Taoism traditional values, Confucius thought, and the Buddhism spirit of volunteerism and trust in social harmony is practical for ecology protection. In summary, integrating social norms, civic reciprocity, cooperatives, and social networks and connections with various stakeholders for environmental protection investment produce sound results. According to the innovation and diffusion theory, several factors challenge the promotion and diffusion of innovation and new technology like CrA technology. Adoption is constrained by time and actions^61^. CrA is both an industrial science and a technological product design, which needs persuasion and acceptance antecedent to dissemination. The foundation of technology acceptance model components, perceived usefulness, and perceived easiness of new technology determine information technology users’ acceptance^36^. Moreover, the theory of planned behavior assumes attitude and behavior influence personal disposition, acceptance, and behavior^62^. Attitude and perceived behavior determine the likability probability regarding a given behavior.

Thus, CAM needs acceptance by users. Studies by social psychologists show that social capital and trust disposition relates to the behavioral intention^63^; moreover, the institutional trust had a significant impact on consumer trust in food, faith in environmental attributes, nanotechnology, and functional foods^64^. In CrA development, understanding traditional values, culture, trust, and belief is important. There is a long tradition in Chinese religion and philosophy, such as Taoism, that enables ecological security^65^. Taoism accords well with ecology and sustainable agricultural development^66^. The wisdom of Taoism and Confucianism is deemed to be essential for economic prosperity and sustainability^67^. Buddhism claims that economic security is in equilibrium with ecological sustainability^68^, while Confucianism is about living in harmony with nature^69,70^.

In psychology and behavioral economics, both social capital and moral values affect human decisions about the adoption of agricultural waste recycling technologies^71^. Empirical studies confirmed that behavior affects learning decisions, and that farmers’ attitude towards the environment affects agricultural practices^72^. In the context of analysis, showed that economic incentives increase the adoption of conservation practices in Indiana^73^. Other studies consider households’ e-waste recycling behavior in China^35^, with focus on different subjects such as young adults^74^, and multi-family dwelling^75^, or on type of waste such as plastic waste^76^. Other examples investigate waste recycling and reuse^35^, as well as attitude and behavior towards the development of waste minimization designs^77^.

This study, thus, considers multiple socio-psychological, behavioral, and technology adoption theories. Understanding the psychological foundation and nudging affects the promotion of CrA. This study reveals the opportunity for future agricultural policy and sustainable development. Farmers’ WTP for waste recycling highlights that agriculture policy design must tackle the growing agriculture waste generation. Undeniably, the outlook of the continued volume of agriculture waste, as well as the growing CAM concerns, is due to increasing population and agriculture production. We found that farmers have a moderate level of understanding of the adverse impact of improper disposal of crop-livestock waste.

In this context, the logit model illustrates the necessity of employing advanced models instead of discrete logit models and considers mixed theories to harness the interwoven features of farmers' volition in involving in pro-environment behavior. Importantly, empirical results reveal how basic characteristics and infrastructure explain agriculture waste recycling, annual income, and farm size affect farmers' WTP for CrA. Our study explores farmers' respective trust in institutions and the government and found indifferent statistical results. We found that Putnam’s social capital has indeterminately affected farmers' WTP amount for CrA. Our study stresses that CrA is novel in modern agriculture; thus, the government should pay attention to integrating it into agriculture production, education, science, and environmental protection. Inheritance of CAM as a systematic approach requires technology, science, specialization, and the best use of wastes as a resource. Our study has limitations. The survey was collected in 2012. However, CrA requires long-term datasets, and government subsidy can be effective in the short but not the long run.

Generally speaking, in fostering CrA, traditional values, religion, morals, and norms should be considered. In developing CrA, first, there must be a common understanding from all stakeholders about the pros and cons. Secondly, CrA needs nudging, as well as a systematic approach in a campaign to teach farmers via traditional instruments, norms, altruism values, morals, manners, and beliefs to demonstrate how the modern cycling accords with traditional recycling resources waste disposal. Farmers' engagement in CAM includes green production, garden farming, and resource optimization, which should be linked with the market and the adoption process.

## Methodology

### Study site

This study was conducted in selected sites of the Hubei Province. China has a total area of 9.6 million square km. China has two unique administrative regions, four municipalities, five autonomous administrative regions, and 34 provinces. The total projected population figure in China is about 1.4 billion population. Hubei is situated in the middle of the Yangtze River. Hubei is located at 29° 05′–38° 20′ North and 108° 21′–116° 07^′^ East and extends across the Yangtze River and Han River. This province covers an area of 185,900 square km, which accounts for 1.94% of the total area of China. It has a mean annual temperature range from 15 to 7 °C, and its yearly rainfall varies from 800 to 1,600 mm, accordingly. Hubei province has subtropical monsoonal climatic conditions. The eastern, western, and northern regions of Hubei province are surrounded by mountains, such as Wuling Mountain, Daba Mountain, Wudang mountain, and Dabie mountain. The Jianghan plain crosses central and southern Hubei province, where the terrain is relatively flat. Hubei is a major base for grain and oil production in China.

### Survey design

A CV approach enables us to measure and determine farming households WTP for CrA technology in Hubei province. CVM is a popular approach applied for non-marketable goods. To gauge farmers' WTP for CrA, this study employed a well fitted double-bound bid in a closed-ended and open-ended questionnaire (see Supplementary Information, Annexure). Antecedent to WTP questions, farmers were educated on environmental and economic benefits, concepts, and practices of CrA. They were then asked, “*how much are you WTP for your contribution to pollution?*” This payment was per month. If the farmers replied “yes,” the bid increased until they answered no. The highest yes response value was recorded as the maximum WTP. If the farmers answered “no,” the bid reduced until the farmers answered yes. The bid choices were provided in this form “A. ≤ 10 yuan, B. 11–20 yuan, C. 21–30 yuan, D. 31–40 yuan, E. 50–60 yuan, F. 61–70 yuan, G. 71–80 yuan, H. 81–90 yuan, I. 91–100 yuan, and J. 101 and above. The CV method suffers from hypothesis bias, such as zero bid and strategic bias^78^. In consideration of this, the researchers used some bias controlling extant methods, such as a pre-survey briefing on the purpose of the study, explaining environmental issues and well-known WTP methods, showing identity cards to farmers to build trust, and conducting face to face interview to avoid non-response.

### Sampling design

Researchers employed a multistage sampling technique. In the first stage, the purposive sampling technique was employed to select Hubei province. Secondly, Hubei province study sites, such as Xinzhou, Suizhou, and Huanggang city were included in the sample. The smallest administrative unit from each county and district samples were considered in the sample frame. Finally, a total of 398 samples were considered from the cluster sample shown in Table 6.

**Table 6:** Sample distribution.

County/city/district	Willing		Not willing		Total	
	#	%	#	%	#	%
Xinzhou district	39	9.8	19	4.8	58	14.6
Suizhou	156	39.2	44	11.1	200	50.3
Huanggang city	105	26.4	35	8.8	140	35.2

All numbers in the table are rounded off.

### Analysis method

Surveyed data were fed into Excel and imported to SPSS 22.0 and Stata 15.0 statistical software to analyze the descriptive statistics and construct the logit and Tobit model.

In terms of the econometric approach, the discrete choice model has been commonly used to determine whether people are willing to pay for public goods. The binary nature of CVM responses calls for a statistical model appropriate for a discrete dependent variable^79^. When an individual is asked to accept or reject a project that generates an environmental improvement from ${z}^{0}$ to ${z}^{1}$, it is important to understand what is the individual’s WTP to the project. However, “yes” or “no” responses only provide qualitative information. Thus, to extract more detailed information on WTP, we need a statistical model that relates individuals’ responses to monetary amounts. In line with Johansson^80^, let us consider an individual that maximizes his utility subject to a budget constraint. Then, the individual's indirect utility function can be written as follows:

1 $$U=u\left[x\left(p,y,z\right),z\right]={u}_{0}\left(p,y,z\right),$$

where *x* is a 1·n vector of private goods, and z is a 1·m vector of environmental goods. The quantity demanded of private goods is a function of prices (p), income (y), and provision of quality environmental commodities (z). The indirect utility function decreases as prices increase, while it increases with income and environmental quality. If environmental quality changes, the change in utility is as follows:

2 $$u={u}_{1}\left(p, y,{z}^{1}\right)-{u}_{0}\left(p,y,{z}^{0}\right)$$

where the superscript 0 (1) denotes initial (final) levels of the environmental good. Since the utility function is not observable, a monetary measure is necessary to evaluate the change in utility. Thus, we consider the concept of CV, which represents the maximum amount of income that can be taken from the individual while leaving her/him just as well off as before the change. More specifically,

3 $$u=u\left(p, y-CV,{z}^{1}\right)=u\left(p,y,{z}^{0}\right).$$

In other words, CV is the individual’s WTP for an improvement in environmental quality. In our specific case, the farmer adopting a CrA technology increases her/his utility as a consequence of the various benefits obtained from its use. This farmer is thus willing to pay an amount of money to secure this utility gain. Following the seminal article by Hanemann^79^, if we assume that the utility function has some unobservable components that are thus treated as stochastic, then the individual's utility function can be written as follows:

4 $$u={u}_{1}\left(y,s,z\right)={u}_{0}\left(y,s,z\right)+{\varepsilon }_{i},$$

where ${\varepsilon }_{0}$ and ${\varepsilon }_{1}$ are identically and independently distributed random variables with zero means. The individual's response is a random variable with cumulative WTP distribution j. Therefore, the probability of a positive answer is the probability that the individual thinks that she/he is better off, even given the required payment^24^; thus, u_1_ > u_0_. For respondent j, the probability is

5 $${\rm Prob}\left\{\text{"yes}" to j \right\}=Pr \left({u}_{1}(WT{p}_{j}-p, zj,{\varepsilon }_{1}j\right)>{u}_{0}\left(j,zj, {\varepsilon }_{0}j \right).$$

When CrA_WTP_ (j) is the standard normal *CDF*, a logit model is obtained.

The use of open-ended questions brings about a standard problem in contingent valuation studies, that is that respondents frequently state zero WTP. It is worth noting that “protest zero” is usually defined as a zero WTP response that is given to protest against a particular aspect^81^. Therefore, a dichotomous double open-bid has traditionally been addressed using a maximum likelihood censoring at zero values. Failure to recognize the censored or truncated distribution of bids in open-ended contingent valuation surveys results in biased and inconsistent parameter estimates. A straightforward solution is the Tobit model, which explicitly accounts for the fact that WTP values are censored at zero. The Tobit model can be specified as follows ^82^^:^

6 $$WTP{i}^{*}={X}^{^{\prime}}\beta +{\varepsilon }_{i}, \,\,WTPi*=\left\{\begin{array}{c}WT{P}_{1}\\ WT{P}_{2}\\ \begin{array}{c}\vdots \\ WTP{i}_{i}\end{array}\end{array}\right\},\,\,x=\left\{\begin{array}{c}{x}_{1} {x}_{2} \dots {x}_{1m}\\ {x}_{21} {x}_{22} \dots {x}_{2m}\\ \begin{array}{c}\begin{array}{ccc}\vdots & \vdots & \begin{array}{cc}\vdots & \vdots \end{array}\end{array}\\ {x}_{n1} {x}_{n2} \dots {x}_{nm}\end{array}\end{array}\right\},\,\,\beta =\left\{\begin{array}{c}{\beta }_{0}\\ {\beta }_{1}\\ \begin{array}{c}\vdots \\ {\beta }_{n}\end{array}\end{array}\right\}\,\,and\,\,\varepsilon =\left\{\begin{array}{c}{\varepsilon }_{1}\\ {\varepsilon }_{2}\\ \begin{array}{c}\vdots \\ {\varepsilon }_{i}\end{array}\end{array}\right\}$$

7 $$WTP{i}^{*}={x}^{^{\prime}}\beta +{\varepsilon }_{i},\varepsilon |x\sim Normal(0,{\sigma }^{2})$$

8 $$WTPi={\rm max}(0,WTP{i}^{*})$$

where $WTP{i}^{*}$ is the unobserved continuous dependent variable, ${X}^{^{\prime}}$ is a set of independent variables, and ${\varepsilon }_{i}$ is assumed to be i.i.d. disturbance term with N (0,$\sigma )$. Thus, the observed WTP variable can be defined as:

9 $$\left\{ {\begin{array}{*{20}l} {WTPi^{*} = 1} \hfill & {if\,WTPi^{*} > 0} \hfill \\ {WTPi^{*} = 0 } \hfill & {if\, WTPi^{*} \le 0} \hfill \\ \end{array} } \right.$$

The WTP amount is censored at zero due to all positive zeros and negative values of $WTP{i}^{*}$ observed close to zero.

### Conceptual model and hypothesis

**Figure 5:**
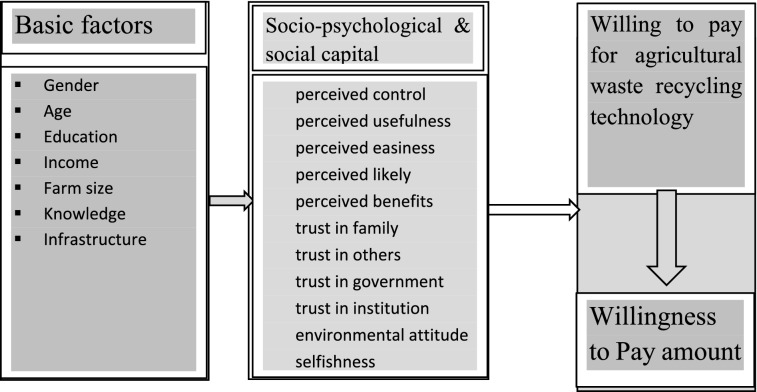
Conceptual framework (author’s elaboration).

Moving to our context of analysis, the dependent variable defined above captures farmers' willingness to pay for agricultural waste recycling technologies. The conceptual hypotheses are listed in Fig. 5. Based on the existing literature, it is assumed that altruism values positively influence farmers’ view of the environment, which, in turn, is positively related to the awareness of the consequences in terms of agricultural waste usage and reliance on linear “take-make-dispose” agricultural practices. Auguste Comet, believed to be a founder of altruism, confirmed that social ties avoid ego and make people unselfish, which accords with the definition for selflessness^27^; selflessness starts from self-benefiting, and it is gradually motivated by mutual benefiting, sharing, comforting cooperation and ends in seeking others’ welfare. Altruism thought presumes individuals that influence other individuals towards environmental sustainability. Altruism encompasses the presupposition to pre-alerted individuals and educators to change the mind of others towards environmental wellbeing^83^. Altruism affects individuals' ecological thinking and recycling behavior. According to planned behavior, perception, attitude, and perceived behavioral control influence recycling and sustainability behavior. As noted, basic household information is hypothesized to affect farmers' psychology and WTP for CrA directly or indirectly. According to Wang^35^ economic conditions, benefits, and returns influence recycling behavior. Thus, recycling technology’s immediate financial gains positively influence recycling behavior regarding WTP. Putnam’s norm reciprocity and institutional repository influence farmers' WTP for CrA. Agricultural waste management and environmental pollution control is a moral project. The opposite of altruism is considered in this study. The detail description of variables for polluter farmers pay presented in Table 7.

**Table 7:** Variables and descriptions.

Variables	Description	Mean	SD
Gender	Gender of householder (1 = male, 0 = otherwise	0.658	0.475
Age	Age in years	51.985	0.529
Education	(1 = illiterate, 2 = primary, 3 = Junior, 4 = Highschool, 5 = College, and above)	2.716	0.940
Farmsiz	Farm size in mu	1.442	0.822
Income	Annual income in yuan	3.105	2.545
Infrast	Infrastructure access (1 = access, 0 = otherwise	0.716	0.451
Kca	Knowledge of CrA (1 = know, 0 = otherwise)	0.437	0.497
Trusto	Trust-in-neighbors (1 = SD, 2 = D, 3 = G, 4 = agree, 5 = SA)	2.815	1.084
Trustf	Trust-in-family 1 = SD, 2 = D, 3 = G, 4 = agree, 5 = SA)	2.982	1.195
Perceivc	Perceived control (1 = SD, 2 = D, 3 = G, 4 = agree, 5 = SA)	2.872	1.152
Perceivl	Perceived likely (1 = SD, 2 = D, 3 = G, 4 = agree, 5 = SA)	3.199	1.227
Attlab	Labor attitude (1 = SD, 2 = D, 3 = G, 4 = agree, 5 = SA)	2.889	1.318
Atthi	Health attitude (1 = SD, 2 = D, 3 = G, 4 = agree, 5 = SA)	1.734	0.774
Intentr	Recycling intention (1 = SD, 2 = D, 3 = G, 4 = agree, 5 = SA)	1.829	0.864
Intente	Environmental intention (1 = SD, 2 = D, 3 = G, 4 = agree, 5 = SA)	1.889	0.940
Behavs	Sustainability behavior (1 = SD, 2 = D, 3 = G, 4 = agree, 5 = SA)	2.131	0.985
Behavr	Recycling behavior (1 = SD, 2 = D, 3 = G, 4 = agree, 5 = SA)	2.319	1.107
Pu	Perceived usefulness (1 = SD, 2 = D, 3 = G, 4 = agree, 5 = SA)	2.837	1.125
Puertu	Perceived returns (1 = SD, 2 = D, 3 = G, 4 = agree, 5 = SA)	2.388	1.012
Envirop	Environmental Perception (1 = SD, 2 = D, 3 = G, 4 = agree, 5 = SA)	1.827	0.877
Peu	Perceived easiness (1 = SD, 2 = D, 3 = G, 4 = agree, 5 = SA)	2.415	1.093
Trustin	Trust-in-institutions (1 = SD, 2 = D, 3 = G, 4 = agree, 5 = SA)	3.085	1.161
Trustg	Trust-in-government (1 = SD, 2 = D, 3 = G, 4 = agree, 5 = SA)	2.590	1.303
Altrusm	Environmental selfishness (1 = SD, 2 = D, 3 = G, 4 = agree, 5 = SA)	2.889	1.191

*SD* strongly, *SA* strongly disagree.

## Supplementary information

Supplementary information
