# Supplementary material for: Cognition of agriculture waste and payments for a circular agriculture model in Central China
Source: Sci Rep. 2020 Jul 2;10:10826. doi: 10.1038/s41598-020-67358-y (PMC7331619; doi:10.1038/s41598-020-67358-y)
Supplement: Supplementary file 1 — Supplementary information [file 41598_2020_67358_MOESM1_ESM.docx]

**Annexure**

## The survey included five categories of questions which are explained below.

## Necessary householder information on CrA: It infers the implication of CAM

## Infrastructure and environmental conditions regarding waste facilities: It ascertains whether there are roads, water, market, landline, wired teleservice, and waste facilities, such as treatment and disposal facilities.

## Knowledge of agricultural waste and environment: It assesses the impact of farmers' evaluation of crop residues, manure, and other farming wastes on the environment.

## Environmental awareness, perception, and attitude: This includes farmers’ perception of recycling behavior or intention to perform an act, originates from their environmental comprehension.

## The implication of social capital, such as institutional trust, interpersonal trust, norm reciprocity, and repository correlation with CrA technology usage.

## Understanding CrA concepts, practices, and principles, such as agriculture waste recycling technology, CE, two-type society, cleaner production, green production, and sustainable development.

## Willingness-to-pay: This section was the focus of the contingent valuation. It focused on whether farmers are willing to pay and how much they will pay in yuan per month.
